# Usage of the Heterologous Expression of the Antimicrobial Gene *afp* From *Aspergillus giganteus* for Increasing Fungal Resistance in Olive

**DOI:** 10.3389/fpls.2018.00680

**Published:** 2018-05-23

**Authors:** Isabel Narvaez, Titouh Khayreddine, Clara Pliego, Sergio Cerezo, Rafael M. Jiménez-Díaz, José L. Trapero-Casas, Carlos López-Herrera, Isabel Arjona-Girona, Carmen Martín, José A. Mercado, Fernando Pliego-Alfaro

**Affiliations:** ^1^Instituto de Hortofruticultura Subtropical y Mediterránea “La Mayora", Departamento de Biología Vegetal, Consejo Superior de Investigaciones Científicas, Universidad de Málaga, Málaga, Spain; ^2^IFAPA Centro de Churriana, Málaga, Spain; ^3^Departamento de Agronomía, College of Agriculture and Forestry, Universidad de Córdoba, Campus de Excelencia Internacional Agroalimentario, Edificio C-4 Celestino Mutis, Córdoba, Spain; ^4^Instituto de Agricultura Sostenible, Consejo Superior de Investigaciones Científicas, Campus de Excelencia Internacional Agroalimentario, Córdoba, Spain; ^5^Departamento de Biotecnología-Biología Vegetal, ETS Ingeniería Agronómica, Alimentaria y de Biosistemas, Universidad Politécnica de Madrid, Madrid, Spain

**Keywords:** fungal resistance, *Olea europaea*, transgenic plants, *Rosellinia necatrix*, *Verticillium dahliae*, verticillium wilt, white root rot

## Abstract

The antifungal protein (AFP) produced by *Aspergillus giganteus*, encoded by the *afp* gene, has been used to confer resistance against a broad range of fungal pathogens in several crops. In this research, transgenic olive plants expressing the *afp* gene under the control of the constitutive promoter CaMV35S were generated and their disease response against two root infecting fungal pathogens, *Verticillium dahliae* and *Rosellinia necatrix*, was evaluated. Embryogenic cultures derived from a mature zygotic embryo of cv. ‘Picual’ were used for *A. tumefaciens* transformation. Five independent transgenic lines were obtained, showing a variable level of *afp* expression in leaves and roots. None of these transgenic lines showed enhanced resistance to Verticillium wilt. However, some of the lines displayed a degree of incomplete resistance to white root rot caused by *R. necatrix* compared with disease reaction of non-transformed plants or transgenic plants expressing only the GUS gene. The level of resistance to this pathogen correlated with that of the *afp* expression in root and leaves. Our results indicate that the *afp* gene can be useful for enhanced partial resistance to *R. necatrix* in olive, but this gene does not protect against *V. dahliae*.

## Introduction

Olive (*Olea europaea* L.), the only species producing edible fruits within the *Oleaceae* family, is one of the most ancient cultured plants probably domesticated from wild olives (*O. europaea* L. subsp. s*ylvestris*) around 6000–5000 BC (López-Escudero and Mercado-Blanco, 2011). Olive is one of the most important fruit crop in the Mediterranean basin, with Spain and Italy being the most important producer countries worldwide (FAOSTAT, 2014). Most olive fruit production is used for olive oil, this crop ranking sixth in the world’s production of vegetable oils (Conde et al., 2008). Olive oil is a main component of the Mediterranean diet having a high nutritional value due to its elevated content in secondary metabolites and unsaturated fatty acids (Pérez-Jiménez et al., 2007; Matthäus and Özcan, 2011).

In recent years, olive cultivation has been seriously threatened due to attack by a number of pathogens that significantly compromise its production. *Verticillium dahliae* Kleb., the causal agent of the Verticillium wilt disease, is considered the main soil-borne pathogen threatening olive production worldwide (López-Escudero and Mercado-Blanco, 2011; Jiménez-Díaz et al., 2012; Keykhasaber et al., 2017). This fungus causes two disease syndromes, namely defoliating (D) and non-defoliating (ND) (Navas-Cortés et al., 2008; Jiménez-Díaz et al., 2012). The D syndrome is characterized by early drop of asymptomatic green leaves from individual twigs and branches, which eventually leads to their complete defoliation and necrosis. The ND syndrome includes two forms differing in symptom’s severity, namely the acute form, also known as apoplexy, and slow decline. Apoplexy consists of the rapid death of branches, or the entire plant in young trees, the necrotic leaves remaining attached to the symptomatic shoots. Slow decline is recognized mainly by flower mummification and necrosis of inflorescences together with chlorosis and necrosis of leaves that develop on individual branches.

White root rot caused by the fungus *Rosellinia necatrix* is also a common disease of fruit trees that can be particularly damaging in the Mediterranean area because of prevailing favorable environment for the development of both, the fungus and susceptible crops (Sztejnberg et al., 1980; Pliego et al., 2012). This ascomycete attacks a large number of woody and semi-woody plants, and it has also been found in bulbs and rhizomes (Pérez-Jiménez, 2006). *Rosellinia necatrix* invades the plant through the root system forming typical white mycelial fans. Later, the white mycelium turns greenish-gray or black, forms plaques on the bark and invades the whole root system causing a generalized rotting of tissues. The symptoms in the aerial part can evolve either quickly or slowly, leading to wilting of leaves, death of branches, and eventually death of the tree. This fungus has been isolated from olive roots in France (Guillaumin et al., 1982), Portugal (Roca et al., 2016), and Spain (García Figueras and Celada Brouard, 2001). According to Roca et al. (2016), soil solarization and treatment with fluazinam fungicide are the best measures for the management of *R. necatrix* in olive.

The use of resistant genotypes is the best approach for control of fungal diseases in woody plants. In olive, some cultivars have been identified that are highly resistant to the ND pathotype of *V. dahliae*, e.g., ‘Changlot Real,’ ‘Empeltre,’ ‘Frantoio,’ ‘Oblonga,’ and ‘Koroneiki’ (Jiménez-Díaz et al., 2012). However, the most popular and economically important olive cultivars, e.g., ‘Arbequina,’ ‘Hojiblanca,’ and ‘Picual’ are susceptible or extremely susceptible (López-Escudero and Mercado-Blanco, 2011). Moreover, cultivars resistant to the ND pathotype are highly susceptible to the D one (López-Escudero et al., 2004) and conventional breeding for Verticillium resistance in olive has given rise to limited results (López-Escudero and Mercado-Blanco, 2011). As far as we know, evaluation of white root rot resistance in commercial olive cultivars has not been addressed yet.

Genetic transformation can be a useful approach for the development of resistance against several plant pathogens. Increased resistance against fitopathogenic fungi has been achieved in several plant species through overexpression of plant antifungal genes, such as those encoding chitinase or glucanase (Ceasar and Ignacimuthu, 2012). However, main limitations of this strategy are the low level of resistance achieved with the introduction of a single gene and the narrow range of pathogens to which resistance is acquired (Dana et al., 2006). The use of genes from fungi or bacteria encoding for antifungal activities, such as those isolated from *Trichoderma harzianum* encoding for the antimicrobial (AM) proteins chitinase and glucanase, has proven to be a more successful strategy (Lorito et al., 1998; Bolar et al., 2001; Mercado et al., 2015). These AM proteins protect higher eukaryotes against invading pathogens, whereas in lower eukaryotes and prokaryotes they seem to have a role in competition for nutrients between species that share the same ecological niche (Hegedüs and Marx, 2013). The mold *Aspergillus giganteus* produces a small, basic and cysteine-rich AM protein that allows for antagonism against filamentous fungi. This protein, named antifungal protein (AFP), is secreted as a 91-amino acid inactive precursor containing a signal sequence for secretion and a prosequence that is removed by a protease during the process of secretion (Wnendt et al., 1994; Martínez-Ruiz et al., 1997). The prosequence maintains the protein inactive until it has crossed the plasma membrane. The mature AFP protein contains 51 amino acids and its structure is similar to the plant defensins and υ-thionins (Campos-Olivas et al., 1995; Lacadena et al., 1995). The antifungal mechanism of AFP is not fully understood. The AFP protein disturbs the integrity of the plasma membrane and inhibits chitin biosynthesis in sensitive fungi (Leiter et al., 2017). Additionally, AFP may enter the host cell, binding to fungal nucleic acids and promoting charge neutralization and condensation of DNA (Martinez Del Pozo et al., 2002; Moreno et al., 2006). However, it is not yet clear whether the binding affinity of AFP to DNA contributes to its antifungal activity.

The expression of the *afp* gene in transgenic plants enhanced resistance to diseases in rice (Coca et al., 2004; Moreno et al., 2005), wheat (Oldach et al., 2001; Li et al., 2008) and pearl millet (Girgi et al., 2006) caused by a broad range of fungal pathogens, e.g., *Magnaporthe grisea*, *Fusarium* spp., *Blumeria* (*Erysiphe*) *graminis* f. sp. *graminis*, *Puccinia recondita*, *Puccinia substriata*, and *Sclerospora graminicola*. The aim of the present study was to investigate if the ectopic expression of the *afp* gene from *A. giganteus* enhances resistance to Verticillium wilt caused by the D *V. dahliae* pathotype and White root rot caused by *R. necatrix* in olive plants. To this purpose, embryogenic cultures derived from a seed of cv. ‘Picual’ were transformed with the *afp* gene and the disease response against both pathogens of the transgenic plants recovered was assessed.

## Materials and Methods

### Plant Material

Olive (*Olea europaea* L.) embryogenic calli, line P1, were induced from an excised radicle of a mature zygotic embryo of cv. ‘Picual,’ following the protocol described by Orinos and Mitrakos (1991). Embryogenic cultures were maintained in olive cyclic embryogenesis medium [ECO basal medium containing ¼ OM macroelements (Rugini, 1984), ¼ MS (Murashige and Skoog, 1962) microelements, ½ OM vitamins and 550 mg/L glutamine] in darkness at 25 ± 2°C and transferred to fresh medium at 4-week intervals (Pérez-Barranco et al., 2009).

### Binary Vector and Olive Transformation

A DNA fragment containing a synthetic *afp* gene fused to a signal peptide from the secreted tobacco AP24 protein (Melchers et al., 1993) was amplified by PCR from the pCubi::synt-afp::nos plasmid (Coca et al., 2004), using the primers 5′-CGATAAGCTTGGATCCGGCCACCATGTCCAACAACATGGG-3′ and 5′-GGTCGGATCCCTATGACTAGCAGTAGCACTTGCCCTTGTAGC-3′. The PCR product was purified with the QIAquick PCR Purification Kit (Qiagen), digested with *BamH*I and inserted into the *BamH*I restriction site of the pBIN61 plasmid, a pBIN19 derived plasmid, under the control of the constitutive promoter CaMV35S. This binary vector, containing the *nptII* gene for plant selection, was introduced into the disarmed *A. tumefaciens* strain AGL1 (Lazo et al., 1991) by the freeze-thaw method (Höfgen and Willmitzer, 1988). *Agrobacterium* cultures were incubated at 28°C in LB medium supplemented with 10 mg/L rifampicin and 50 mg/L kanamycin, at 250 rpm. Before embryogenic callus inoculation, the bacterial suspension was centrifuged at 5000 *g*, the pellet washed with 10 mM MgSO_4_ and finally diluted in ECO medium at 0.5–0.6 OD_600_.

Globular somatic embryos from the embryogenic line P1 were transformed using the protocol described by Torreblanca et al. (2010). The explants were incubated in a diluted *Agrobacterium* culture for 20 min under mild agitation. Then, explants were blotted dried on sterile filter paper and cultured on ECO solid medium in Petri dishes at 25°C in darkness for 2 days. After that, explants were washed with 5 ml ECO liquid medium supplemented with 250 mg/L cefotaxime and timentin at 25°C for 2 h, gently dried on sterile filter paper and cultured on selection medium, i.e., ECO solid medium supplemented with 250 mg/L of cefotaxime, 250 mg/L of timentin, and 200 mg/L of paromomycin. Explants were incubated in darkness for at least 4 months with reculturing on fresh selection medium at weekly intervals during the first month and bi-weekly thereafter. Explants that showed proliferation in the solid selection medium were grown individually in 250 ml culture flasks containing 40 ml of liquid ECO medium supplemented with 250 mg/L of cefotaxime and 50 mg/L of paromomycin (Duchefa Biochemie) in an orbital shaker at 120 rpm for two cycles of 3 weeks. After that, embryogenic calli were filtered through a 2-mm mesh and somatic embryos obtained were used for plant regeneration following the protocol of Cerezo et al. (2011). Briefly, somatic embryos were cultured on maturation ECO medium, basal ECO medium without growth regulators and cefotaxime, supplemented with 1 g/L activated charcoal and 200 mg/L of paromomycin for 8 weeks. Then, maturated embryos were transferred to germination medium (Clavero-Ramírez and Pliego-Alfaro, 1990), modified MS with 1/3 MS macroelements, MS microelements, 10 g/L sucrose and supplemented with 200 mg/L paromomycin, for 3 months under 40 μmol m^-2^ s^-1^ irradiance level. Plantlets were micropropagated in DKW medium (Driver and Kuniyuki, 1984) as modified by Revilla et al. (1996) for 9 weeks, and later acclimatized to *ex vitro* conditions in jiffy trays with peat moss:perlite (1:1). Plants were grown in a confined greenhouse with a cooling system, 30°C maximum temperature, and daylight conditions. Plants recovered from an embryogenic line transformed with pBINUbiGUSInt plasmid (Torreblanca et al., 2010) expressing the GUS reporter gene, as well as those recovered from non-transformed embryogenic calli were used as controls.

### Phenotypical Analysis of Transgenic Plants

To evaluate the *in vitro* behavior of transgenic AFP lines, shoot segments with two nodes and deprived of the shoot apex, approximately 1.5 to 2 cm long, were isolated and cultured on RP medium [DKW macro and micronutrients as modified by Roussos and Pontikis (2002), and vitamins of Roussos and Pontikis (2002)], supplemented with 2 mg/L zeatin riboside (Vidoy-Mercado et al., 2012) for 8 weeks. The number of axillary shoots and their length were measured over three subcultures. To evaluate rooting capacity, 2-cm long apical shoots with one or two nodes were cultured on tubes containing 3 ml of basal RP medium supplemented with 10 mg/L IBA for 3 days. Then, shoots were cultured on RP solid medium without hormones but supplemented with 1 g/L activated charcoal. The number of roots and the length of the main root were measured after 9 weeks. Twenty shoots per independent transgenic line were used and the experiment was performed by triplicate.

Plant height and the diameter of the main stem were measured in 1-year old plants growing in the greenhouse under natural conditions. Fifteen plants per transgenic and control lines were evaluated.

### Molecular Analysis of Transgenic Plants

The transgenic nature of *afp* transgenic plants was confirmed by PCR amplification of a 200-bp fragment from the *afp* gene and a 700-bp fragment from *nptII*. Genomic DNA was isolated from leaves of *in vitro* plants using the protocol of Healey et al. (2014). Primers for PCR assays were F: 5′-TCCTCCTTCGTCTTCTTCCT-3′ and R: 5′-ACTTGCCCTTGTAGCTGTCG-3′ for *afp*, and F: 5′-GAGGCTATTCGGCTATGACTG-3′ and R: 5′-ATCGGGAGCGGCGATACCGTA-3′ for *nptII*. All reactions were prepared in a final volume of 20 μl containing 0.5 μl of genomic DNA and 0.5 μM of each primer. Amplification conditions consisted in 4 min at 95°C, followed by 30 cycles of 45 s at 95°C, 45 s at 59°C, and 1 min at 72°C, with a final extension step of 10 min at 72°C.

Young leaves and roots from control and AFP plants were collected, frozen in liquid nitrogen and kept at -80°C until used for RNA extraction. Root tissue, 2 g, was powdered in liquid nitrogen and RNA extracted as described by Chang et al. (1993). After extraction, RNA was precipitated overnight with 10 M LiCl, washed with ethanol 70% and resuspended in RNase free water. RNA from leaves was extracted with the Spectrum^TM^ Plant Total RNA kit (Sigma-Aldrich) following manufacturer’s instructions. RNA concentration and purity was assessed using NanoDrop ND-1000 spectrophotometer (Nanodrop Technologies, Inc., Montchanin, DE, United States). The integrity of RNA samples was visualized on a 1.5% agarose gel under non-denaturing conditions. To remove genomic DNA, RNA was treated with DNase I recombinant, RNase free (Roche). cDNA synthesis was carried out with iScript^TM^ cDNA Synthesis kit (Bio-Rad), according to the manufacturer’s instructions.

Quantitative real-time PCR (qRT-PCR) was performed using iTaq^TM^ Universal SYBR Green Supermix (Bio-Rad) in a final reaction volume of 20 μl containing 0.5 μM of each primer and 1 μl of diluted cDNA, in a Bio-Rad CFX96^TM^ (Bio-Rad). Primers for *afp* amplification were F: 5′-CCATCTGCAAGTGCTACGTC-3′ and R: 5′-ACTTGCCCTTGTAGCTGTCG-3′. The olive Ubiquitin gene (F: 5′-ATGCAGATCTTTGTGAAGAC-3′; R: 5′-ACCACCACGAAGACGGAG-3′) was used as housekeeping gene for normalization (Gómez-Jiménez et al., 2010). PCR conditions were 30 s at 95°C, 40 cycles of 5 s at 95°C and 30 s at 60°C, followed by a melting curve from 65 to 95°C with 0.5°C increment every 5 s. Relative *afp* expression levels in leaves and roots were calculated using the 2^-ΔΔCT^ method (Livak and Schmittgen, 2001). Three biological replicates, with three technical replicates each, were analyzed, and means ± standard deviations were represented.

### *Verticillium dahliae* Infection Assay

Disease reaction of AFP transgenic lines to Verticillium wilt was assessed as previously described (Jiménez-Fernández et al., 2016; Palomares-Rius et al., 2016) using plants from P1 embryogenic callus (non-transgenic) and transgenic pBINUbiGUSInt (GUS) as controls. A wild olive genotype, StopVert, highly resistant to Verticillium wilt was also included in the assays (Jiménez-Fernández et al., 2016). All plants had been grown in 12 cm diameter plastic pots containing peat moss-perlite substrate at 1:1 ratio with 2 g of osmocote fertilizer, in a confined greenhouse under natural temperature and photoperiod for over 8 months before being used for disease reaction assays.

Monosporic *V. dahliae* isolate V-138 (D pathotype, lineage 1A, race 2; Milgroom et al., 2014; Jiménez-Díaz et al., 2017) was used in initial experiments. For inoculum preparation, 12 disks of 1-week-old V-138 cultures grown on Potato Dextrose Agar (PDA) were transferred to flasks containing 400 g of a sterilized (121°C for 2.5 h) sand:cornmeal:deionized water (9:1:2, w/w) mixture (AMA). Cultures were incubated at 24 ± 1°C in the dark for 1 month, shaking the flasks at 7 days intervals to facilitate the homogeneous colonization of the substrate by the fungus. Then, the infested AMA was mixed with a pasteurized soil mixture (sand:peat moss, 2:1 v/v) (27.5% water holding capacity) at a rate of 10% (v/v). Inoculum density of *V. dahliae* in the infested soil mixture was estimated by serial dilutions on agar plates supplemented with 30 μg/L of aureomycin (AAAu) incubated at 24 ± 1°C in the dark for 7 days. Mean density of inoculum in the soil mixture was 3.8 × 10^7^ colony forming units (cfu) per g of soil.

For inoculation, 10-month-old plants were uprooted and the root system washed free of soil. Plants with the bare roots were transplanted to 13 cm × 13 cm × 12 cm disinfested plastic pots containing the infested soil mixture. Non-inoculated control plants were transferred to a soil mixture containing sterile AMA. After transplanting, plants were grown in a greenhouse adjusted to 16–26°C (mean: 19.7°C) and 55–80% relative humidity (RH) (mean 61%) and a 14-h photoperiod of fluorescent light of 360 μmol m^-2^ s^-1^ until most of them were dead by 63 days after inoculation. Plants were watered regularly and fertilized every 3 weeks with Hoagland’s nutrient solution.

Disease reaction of inoculated and control plants was assessed by monitoring the severity of foliar symptoms on individual plants on a 0 to 4 rating scale according to the percentage of plant tissue affected by leaves chlorosis, leaves and shoots necrosis or defoliation (0 = no symptoms; 1 = 1–33%, 2 = 34–66%, 3 = 67–100%, 4 = dead plant) at 3–4 days intervals. Disease ratings were plotted over time to obtain disease progress curves and the areas under the disease progress curve (AUDPC) were also calculated as described by Campbell and Madden (1990). At the end of the experiment, isolations on AAAu were carried out from shoot segments of inoculated plants to confirm infection by the pathogen.

### *Rosellinia necatrix* Infection Assay

*Rosellinia necatrix* assays were performed as described by Ruano Rosa and López Herrera (2009), using wheat seeds colonized by a single isolate of the fungus (*Rn*400) as inoculum. The wheat grains were soaked in distilled water for 24 h, autoclaved at 121°C and 0.1 MPa for 40 min and inoculated with disks from colonies of *Rn*400 isolate, growing on PDA medium. The seeds were incubated for 15 days at 24°C in the dark. Ten-month old plants growing in the same substrate used for acclimatization were inoculated with colonized wheat seeds (1.05 g/L) and grew in greenhouse conditions at 25°C for 2 months.

Visual symptoms were observed twice a week, using the following scale: (1) healthy plant; (2) leaf chlorosis; (3) first symptoms of wilted and roll up/curl up in the leaves; (4) wilted plant with first symptoms of leaf desiccation; (5) dead plant. AUDPC were calculated as described by Campbell and Madden (1990) and the different tolerance of transgenic olive lines was evaluated using a completely randomized experimental design, using 10 inoculated plants and 4 non-inoculated plants per line.

### Statistical Analysis

Data were subjected to analysis of variance (ANOVA) using SPSS software version 23. The Levene test for homogeneity of variances was performed prior to ANOVA, and multiple mean comparisons were done by LSD. Kruskal–Wallis test was used for mean separation in case of non-homogeneous variances. All tests were performed at *P* = 0.05.

## Results

### Generation of Transgenic AFP Olive Plants

More than 2400 globular somatic embryos (SE) from line P1 were inoculated with the *A. tumefaciens* AGL1 disarmed strain carrying the pBIN61 binary vector, in two independent experiments. After 2 months of selection in a medium supplemented with 200 mg/L paromomycin, all non-*Agrobacterium* inoculated embryos were necrotic while 94 inoculated explants showed some sectors of new callus growth. These calli were cultured individually in liquid ECO medium supplemented with 50 mg/L paromomycin for two cycles of 3 weeks. Then, calli were filtered and SE suspensions plated on solid selection medium. Thirty-six callus lines showed a good proliferation rate after this additional selection phase, which represents a transformation rate of 1.5% based in the tolerance to paromomycin. Each callus line was considered an independent transgenic line.

**FIGURE 1:**
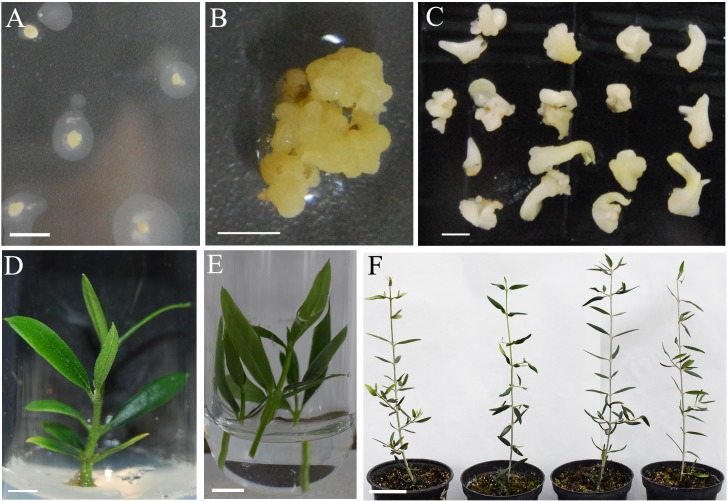
Transformation of olive somatic embryos with *afp* gene from *Aspergillus giganteus*. **(A)** Globular somatic embryos after 2 days of inoculation with *A. tumefaciens*. **(B)** Transgenic *afp* callus growing in selection ECO medium supplemented with 200 mg/L paromomycin. **(C)** Transgenic *afp* somatic embryos after the maturation phase. **(D)** Micropropagated shoot from AFP9 line. **(E)** Transgenic AFP9 shoots in liquid medium with 10 mg/L IBA for rooting. **(F)** From right to left, acclimated plants derived from non-transformed P1 line and transgenic AFP7, AFP9 and AFP10 lines, after 1 year of growth in the greenhouse. Bars correspond to 0.5 cm in **(A–E)** and 5 cm in **(F)**.

For transgenic plant recovery, SE from selected transgenic callus lines were maturated following the procedure of Cerezo et al. (2011) and later germinated in the medium of Clavero-Ramírez and Pliego-Alfaro (1990). Plants from six independent callus lines were recovered. These plants were micropropagated in DKW medium as modified by Revilla et al. (1996) and later acclimatized to greenhouse conditions (**Figure 1**).

### Molecular Analysis of AFP Plants

**FIGURE 2:**
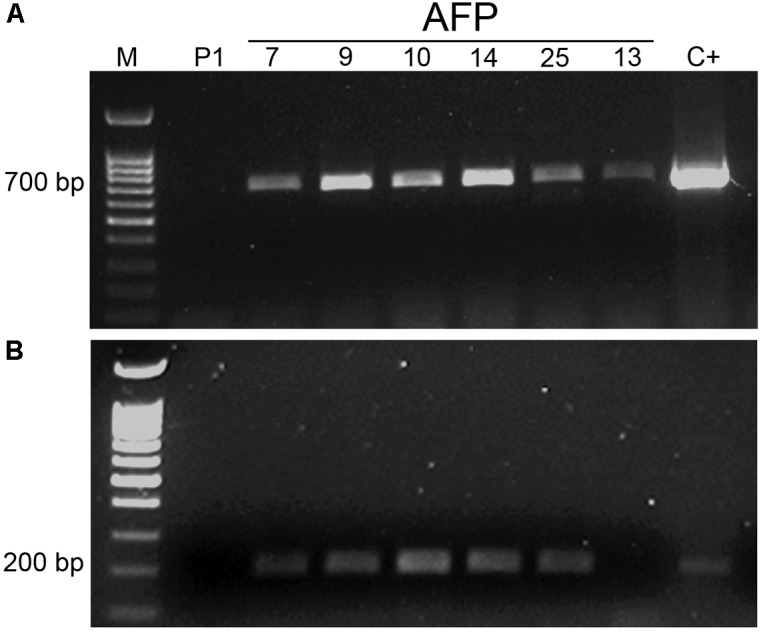
PCR analysis of *afp* transgenic olive plants. **(A)** Amplification of a 700 bp DNA fragment from *nptII* gene. **(B)** Amplification of a 200 bp DNA fragment from the *afp* gene. M: molecular weight marker; C+: pBIN61 binary plasmid; P1: DNA from non-transformed P1 line; AFP: DNA from transgenic *afp* lines.

The transgenic nature of AFP plants was confirmed by PCR amplification of both, *nptII* and *afp* genes (**Figure 2**). DNA from the non-transgenic control plants did not show any PCR amplification. The PCR signal corresponding to amplification of the 700-bp fragment from the *nptII* gene was detected in all transgenic lines (**Figure 2A**). Regarding the *afp* gene, all transgenic lines except line AFP13 yielded the 200-bp DNA band corresponding to amplification of the *afp* gene fragment (**Figure 2B**). The absence of *afp* gene in line AFP13 was confirmed when using the set of PCR primers employed for quantification of *afp* expression by qRT-PCR, which amplified a 75 bp fragment (results not shown).

**FIGURE 3:**
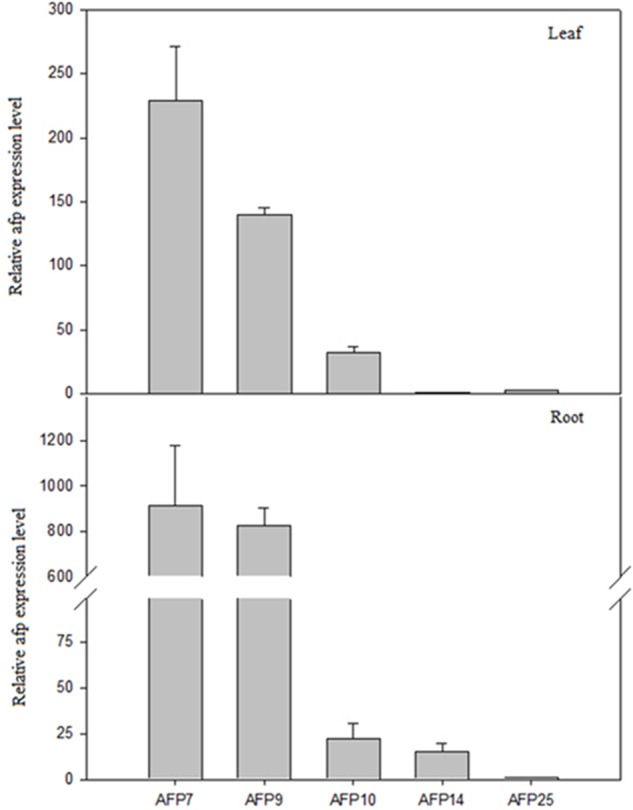
qRT-PCR analysis of *afp* gene expression in leaves and roots of transgenic olive plants. Quantification was based on Ct values as described in Section “Materials and Methods.” The mRNA values were relative to the lowest values obtained in leaves and roots, AFP14 and AFP25, respectively, which were assigned an arbitrary value of 1. Data correspond to mean ± SD of three independent experiments.

The expression of *afp* gene in leaves and roots of transgenic plants was estimated by qRT-PCR (**Figure 3**). The highest levels of transgene expression in both tissues were detected in line AFP7 followed by line AFP9. Line AFP10 showed moderate amount of *afp* mRNA in leaves but low levels in roots. Finally, AFP14 and AFP25 showed the lowest *afp* expression in both organs. As expected, *afp* mRNA was not detected either in controls or line AFP13, where the transgene had not been incorporated, as previously indicated.

### Phenotypical Characterization of Transgenic AFP Plants

The *in vitro* behavior, as well as shoot length and stem diameter of 1-year-old acclimatized AFP plants were assessed to determine the effect of constitutive expression of the *afp* gene in olive growth. For the *in vitro* measurements, stem segments with two lateral nodes were cultured in RP proliferation medium and the developed shoots were evaluated after 8 weeks of culture (**Table 1**). In general, there were minor differences in the number of shoots per explant and the length of the main shoot between transgenic lines and the control. Proliferated shoots were exposed to IBA for 3 days and transferred afterwards to basal RP medium for root emergence. More than 88% of the control shoots rooted, producing 2.4 roots per explant (**Table 1**). The percentage of rooted explants was also similar in all transgenic lines. Line AFP7 produced a lower number of roots per explant than the rest of genotypes. The main root length varied greatly among the different genotypes, being larger in the control non-transformed line and shorter in AFP25 and GUS lines. When 1-year-old plants grown in the greenhouse were evaluated, no significant differences were found in the shoot length and stem diameter between controls and transgenic AFP lines (**Table 2**).

**Table 1:** *In vitro* characterization of transgenic AFP olive plants.

Genotype	Shoots per explant	Length of main shoot (cm)	Rooted explants (%)	Roots per explant	Length of main root (cm)
Control	2.1 ± 0.7^ab^	4.8 ± 1.8^b^	88.9	2.4 ± 1.4^bc^	2.6 ± 1.3^a^
GUS	1.9 ± 0.5^b^	4.5 ± 1.9^b^	75.0	2.4 ± 1.5^bc^	1.2 ± 0.9^bc^
AFP7	2.0 ± 0.5^ab^	4.8 ± 1.2^b^	95.0	1.8 ± 0.8^c^	2.2 ± 1.3^a^
AFP9	2.3 ± 0.7^a^	5.3 ± 1.7^ab^	84.2	3.4 ± 1.8^ab^	1.9 ± 0.9^ab^
AFP10	2.2 ± 0.6^ab^	4.5 ± 1.0^b^	91.9	3.4 ± 1.2^a^	1.7 ± 1.3^abc^
AFP14	2.0 ± 0.5^ab^	5.5 ± 1.3^a^	94.1	2.7 ± 1.3^bc^	1.9 ± 1.3^abc^
AFP25	2.0 ± 0.5^ab^	5.0 ± 1.3^b^	87.1	3.2 ± 1.4^ab^	1.2 ± 0.7^c^

*Data correspond to mean ± SD. Within each column, means with different letters are significantly different by Kruskal–Wallis test at P = 0.05.*

**Table 2:** *Ex vitro* characterization of transgenic AFP olive plants.

Genotype	Shoot length (cm)	Diameter of stem (cm)
Control	35.3 ± 7.9^a^	0.30 ± 0.04^ab^
GUS	43.6 ± 11.4^a^	0.30 ± 0.03^ab^
AFP7	43.5 ± 15.1^a^	0.33 ± 0.06^a^
AFP9	40.6 ± 7.4^a^	0.30 ± 0.04^ab^
AFP10	44.0 ± 7.8^a^	0.33 ± 0.04^a^
AFP14	46.5 ± 9.9^a^	0.32 ± 0.05^ab^
AFP25	40.9 ± 11.7^a^	0.27 ± 0.04^b^

*Data correspond to mean ± SD. Within each column, means with different letters are significantly different by Kruskal–Wallis test at P = 0.05.*

### *Verticillium dahliae* Infection Assays

No symptoms occurred in non-inoculated plants. Plants of non-transformed P1 line as well as those of GUS and AFP transgenic lines showed symptoms characteristic of D pathotype (Navas-Cortés et al., 2008; Jiménez-Díaz et al., 2012) (results not shown) although some differences were observed in their development (see below). As expected, the wild olive resistant genotype StopVert did not show any disease symptom.

**FIGURE 4:**
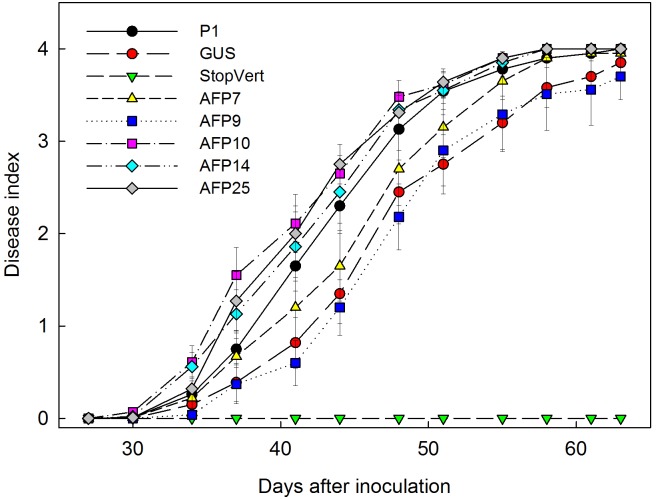
Progress of Verticillium wilt symptoms over time in controls and transgenic olive plants transformed with *afp* gene from *Aspergillus giganteus*. P1: control non-transformed plants; GUS: pBINUbiGUSInt transformed plants, StopVert: wild olive resistant to *Verticillium*; AFP: *afp* transgenic lines. Values correspond to mean ± SE.

**FIGURE 5:**
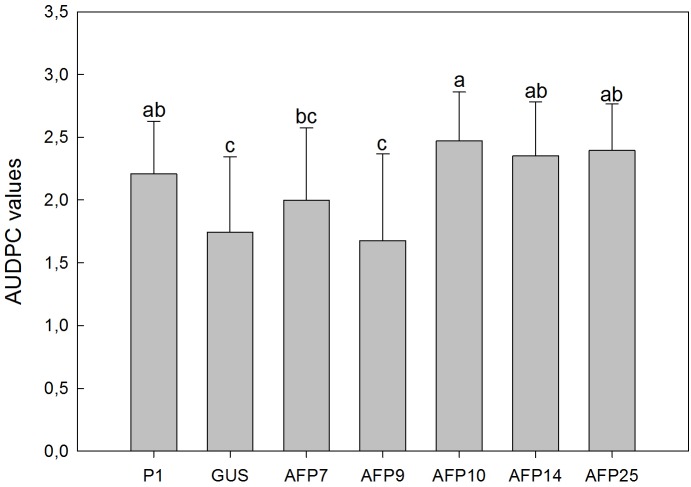
Average values of area under the disease progress curve (AUDPC) in controls and transgenic olive plants inoculated with *Verticillium dahliae*, defoliant strain V-138. P1: control non-transformed plants; GUS: pBINUbiGUSInt transformed plants; AFP: *afp* transgenic lines. Data represent mean ± SD of 10 plants. Columns with different letters indicate significant differences by LSD test at *P* = 0.05.

Verticillium wilt symptoms in plants of lines P1, GUS and *afp*-expressing AFP7, 9, 10, 14, and 25 started to develop by 32 days after inoculation and progressed steadily over time with minor differences. By 63 days after inoculation all plants of lines P1 and AFP7, 10, 14, and 25 were dead, and lines AFP9 and GUS were affected with 100% incidence and a mean severity of symptoms of 3.7 and 3.9, respectively, within the 0–4 rating scale (**Figure 4**). Disease reaction of *afp*-expressing lines, as indicated by AUDPC values, was similar to that in the non-transformed P1 and transgenic GUS lines, independently of the *afp* mRNA levels (**Figure 5**).

### *Rosellinia necatrix* Infection Assay

**FIGURE 6:**
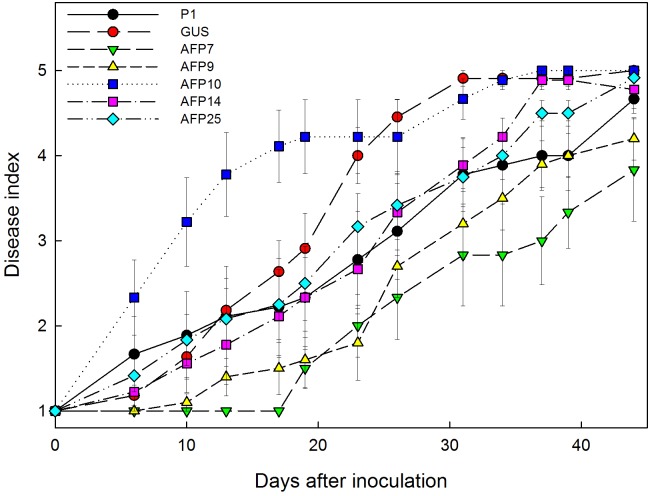
Progress of white root rot symptoms caused by *Rosellinia necatrix* over time in controls and transgenic olive plants transformed with *afp* gene from *Aspergillus giganteus*. P1: control non-transformed plants; GUS: pBINUbiGUSInt transformed plants; AFP: *afp* transgenic lines. Values correspond to mean ± SE.

**FIGURE 7:**
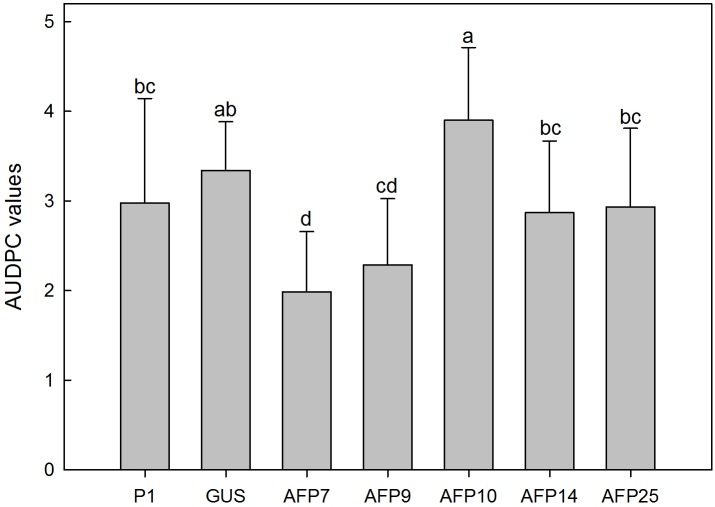
Average values of AUDPC in controls and transgenic olive plants inoculated with *Rosellinia necatrix*. P1: control non-transformed plants; GUS: pBINUbiGUSInt transformed plants; AFP: *afp* transgenic lines. Data represent mean ± SD of 10 plants. Columns with different letters indicate significant differences by LSD test at *P* = 0.05.

No symptoms developed in non-inoculated plants. Disease symptoms in control plants of non-transformed P1 and transformed GUS lines appeared 6 days after inoculation with *R. necatrix* and all plants died by 44 days post-inoculation (**Figure 6**). Transgenic lines AFP7 and AFP9 that showed the highest level of *afp* expression displayed a degree of resistance to white root rot as indicated by an increase in the incubation period and significant reduction in AUDPC values (**Figures 6**, **7**). By contrast, transgenic AFP lines 10, 14 and 25, which showed a low level of *afp* expression, displayed a level of susceptibility to *R. necatrix* similar to that of the control lines. At the end of the experiment, the final mean severity of symptoms was negatively correlated with the level of *afp* expression in roots (Pearson correlation coefficient of -0.968, significant at *P* = 0.01) and leaves (Pearson correlation coefficient of -0.954, significant at *P* = 0.05) of transgenic AFP plants.

## Discussion

In this study, we have investigated whether or not the ectopic expression of *afp* gene from *A. giganteus* in olive increases its resistance to root-infecting fungal pathogens causing different pathogenesis; i.e., root necrosis (necrotrofic *R. necatrix*) and wilting (hemibiotrofic *V. dahliae*). This strategy has successfully been used in other crops, such as rice, wheat and pearl millet (Oldach et al., 2001; Moreno et al., 2005; Girgi et al., 2006; Li et al., 2008) to increase tolerance to diseases caused by different fungi. However, as far as we know, the antifungal effect of AFP against *V. dahliae* and *R. necatrix* has not been evaluated yet, neither in transgenic plants nor in *in vitro* assays using isolated AFP protein (Leiter et al., 2017).

Six independent transgenic olive lines were generated and five of them constitutively expressed the *afp* gene in both roots and leaves. As generally observed in most genetic transformation experiments, the level of transgene expression varied greatly among the different lines. This is due to the influence of the copy number as well as to other factors such as site of integration (Matzke and Matzke, 1998). The expression of *afp* did not affect the *in vitro* behavior or growth in the greenhouse of transgenic plants, as previously observed in other species (Oldach et al., 2001; Coca et al., 2004; Girgi et al., 2006). Similarly, external applications of AFP on plants did not cause any detrimental effects, suggesting that AFP can be safely used in crop protection (Meyer, 2008).

The response of AFP transgenic plants to the two pathogens assayed was completely different. Constitutive expression of *afp* gene in olive enhanced a degree of incomplete resistance against white root rot caused by *R. necatrix*, with the level of *afp* expression being negatively correlated with susceptibility to the disease in the transgenic lines. This relationship agrees with that found in *afp*-transgenic wheat, for which enhanced resistance to powdery mildew (*Blumeria graminis* f. sp. *tritici*) and leaf rust (*Puccinia recondita*) was associated with the level of *afp* gene expression and the low *afp*-expressing lines showed same response as that of the wild type (Oldach et al., 2001).

Contrary to reaction to white root rot, transgenic olive plants expressing the *afp* antifungal gene were as susceptible to the highly virulent D pathotype of *V. dahliae* causing Verticillium wilt as the controls. This is not an unexpected result. Although the antifungal activity of AFP at low concentration has been demonstrated against numerous filamentous ascomycetes, some species showed resistant, suggesting that AFP may operate in a species-specific manner (Theis et al., 2003). The mechanisms of action of AFP seem to rely mainly on disruption of the plasma membrane integrity and inhibition of chitin biosynthesis in sensitive filamentous fungi (Leiter et al., 2017). However, some oomycetes, such as *Phytophthora infestans* and *Sclerospora graminicola*, whose cell wall does not contain chitin, are also susceptible to AFP (Vila et al., 2001; Girgi et al., 2006). AFP_NN5353_, a different antifungal protein produced by *A. giganteus* that shares more than 90% sequence identity with AFP does interfere with cell wall integrity of sensitive fungi, but also increases cytosolic free Ca^2+^, thus disrupting calcium signaling pathways (Binder et al., 2011). The biochemical basis underlying AFP resistance in tolerant fungi are largely unknown. According to Theis et al. (2003), resistance to AFP could be due to lack of a specific interacting partner of AFP in the resistant species or to vacuolar accumulation and degradation of the protein in resistant fungi. Oldach et al. (2001) also observed that the inoculum density influences the response of *afp* transformed wheat to different pathogens, i.e., the enhanced tolerance to *B. graminis* f. sp. *tritici* and *P. recondita* was lost at high doses of inoculum. Further experiments are needed to determine if the lack of resistance to *V. dahliae* in transgenic olive is due to the tolerance of this fungus to AFP protein or to an insufficient level of AFP accumulation in olive tissues.

## Conclusion

Heterologous expression of the *afp* gene from *A. giganteus* can be a useful approach to enhance resistance against some soilborne fungi such as *R. necatrix*. However, this gene does not confer resistance to Verticillium wilt caused by *V. dahliae*, one of the most devastating and widespread olive diseases.

## Author Contributions

IN, TK, and SC were responsible for obtainment, maintenance and *in vitro* characterization of transgenic plants. IN, CP, and CM were responsible for molecular analysis and expression studies of AFP transgenic plants. RJ-D and JT-C performed the *V. dahliae* assays. CL-H and IA-G carried out the *R. necatrix* assays. JM and FP-A planned this research, designed the experiments and wrote the manuscript.

## Conflict of Interest Statement

The authors declare that the research was conducted in the absence of any commercial or financial relationships that could be construed as a potential conflict of interest.
